# Extracts of black bean peel and pomegranate peel ameliorate oxidative stress-induced hyperglycemia in mice

**DOI:** 10.3892/etm.2014.2040

**Published:** 2014-10-30

**Authors:** JIAN-YUN WANG, CHUANG ZHU, TIAN-WEI QIAN, HAO GUO, DONG-DONG WANG, FAN ZHANG, XIAOXING YIN

**Affiliations:** Jiangsu Key Laboratory of New Drug Research and Clinical Pharmacy, Xuzhou Medical College, Xuzhou, Jiangsu 221002, P.R. China

**Keywords:** extract of black bean peel, extract of pomegranate peel, oxidative stress, hyperglycemia

## Abstract

Oxidative stress has a central role in the progression of diabetes mellitus (DM), which can directly result in the injury of islet β cells and consequent hyperglycemia. The aim of the present study was to evaluate the possible protective effects of black bean peel extract (BBPE), pomegranate peel extract (PPE) and a combination of the two (PPE + BBPE) on streptozotocin-induced DM mice. Oxidative stress was assessed by the levels of total antioxidative capability and glutathione in the serum. Fasting blood glucose and insulin levels, as well as the pancreas weight index and the histological changes in the pancreas, were also determined. The results showed that, after fours weeks of treatment with PPE, BBPE or PPE + BBPE, DM mice showed, to different degrees, a decrease in blood glucose, increases in insulin secretion and the pancreas weight index, and an increase in antioxidative activity. These changes were particularly evident in the DM mice subjected to the combined intervention strategy of PPE + BBPE. The histological findings indicated that the injury to the pancreatic islets in DM mice was also ameliorated following treatment. In conclusion, PPE and BBPE, particularly the combination of the two, have the ability to ameliorate hyperglycemia by inhibiting oxidative stress-induced pancreatic damage; this finding may be useful in the prevention and treatment of DM.

## Introduction

Diabetes mellitus (DM) is one of the most frequently occurring chronic diseases worldwide. The condition comprises a group of metabolic diseases characterized by hyperglycemia resulting from aberrant insulin secretion and synthesis. Although the etiology of the disease is not well defined, evidence suggests that oxidative stress or reactive oxygen species (ROS) have a central role in the onset of DM and its complications, which can directly result in the injury of islet β cells and consequent hyperglycemia (1). Hyperglycemia further causes ROS overproduction and exacerbates the pancreatic lesions (2); therefore, antioxidant therapy is a promising treatment strategy for DM that may ameliorate the injury to the function and structure of β cells and regulate the level of blood glucose (3). At present, due to the adverse effects of synthetic hypoglycemic drugs, the use of natural antioxidants, particularly those of plant origin, is attracting considerable focus.

Black bean peel extract (BBPE) has a high concentration of phenolic compounds. The primary phenolic compounds of BBPE are flavonoids, predominantly anthocyanins and proanthocyanidins, which have shown antioxidant (4–6) and antimutagenic activities (7–9) *in vitro* as well as *in vivo*. Xu and Chang (10) found that the seed peel contributed 90% of the total antioxidant capacity of black bean. Pomegranate peel extract (PPE) is also rich in polyphenols, including ellagitannins, gallotannins, ellagic acids, gallagic acids, catechins, anthocyanins, ferulic acids and quercetins (11). These polyphenols exhibit numerous biological activities, such as eliminating free radicals, inhibiting oxidation and the growth of microbes, and reducing the risks of cardio- and cerebrovascular diseases and certain types of cancer (11–14). Due to their strong antioxidant effects, black bean peel and pomegranate peel have been common herbal materials in oriental medicine for hundreds of years (4–6,11). Since diabetes is associated with ROS abnormalities and oxidative stress, it is possible that BBPE or PPE may have a protective effect in DM mice; however, this issue has not been fully discussed.

In the present study, streptozotocin (STZ), an antibiotic cytotoxic to pancreatic β cells due to ROS overproduction-mediated oxidative stress (15–17), was utilized in the production of a DM mouse model. The effects of BBPE, PPE and a combination of the two (PPE + BBPE) on blood glucose, antioxidant parameters, such as the total antioxidative capability (T-AOC) and levels of glutathione (GSH), and the morphological changes in the pancreas of DM mice were observed. The aim of the study was to provide experimental evidence for the clinical application of the two extracts in DM.

## Materials and methods

### Materials

BBPE (containing 40% anthocyanin) and PPE (containing 40% total polyphenols) were supplied by Ningxia Kaiyuan Biotechnology Co. Ltd (Ningxia, China). STZ was obtained from Biomol Research Laboratories, Inc. (Plymouth Meeting, PA, USA) and dissolved in 1% carboxymethyl cellulose (CMC) solution. The T-AOC and GSH kits were purchased from Nanjing Jiancheng Bioengineering Institute (Nanjing, China). The mouse insulin kit was purchased from R&D Systems (Minneapolis, MN, USA). All other reagents were of analytical grade.

### Induction of diabetes in mice and study protocol

Male Kunming mice weighing 20–22 g (Shandong Lukang Animal Pharmaceutical Co. Ltd., Jining, China) were intraperitoneally injected with 150 mg/kg STZ (dissolved in citrate buffer at pH 4.5 immediately prior to injection) to establish a DM model. The mice with a fasting blood glucose concentration of ≥11.1 mmol/l were recognized as successful DM models two days after STZ administration; normal control mice (NS group) received citrate buffer alone. The DM mice were further divided into four groups: i) DM group (n=8; oral administration of 1% CMC solution); ii) PPE group (n=6; oral administration of 400 mg/kg PPE); iii) BBPE group (n=6; oral administration of 400 mg/kg BBPE); and iv) PPE + BBPE group (n=6; oral administration of 200 mg/kg PPE plus 200 mg/kg BBPE). The same volume of CMC solution was administered to the NS group (n=9). The animals were allowed *ad libitum* access to food and water. After four-week treatment via oral gavage, blood samples were collected for the determination of T-AOC and GSH, insulin and blood glucose levels. Following the sacrifice of the animals under sodium pentobarbital anesthesia in order to minimize their suffering, the fresh pancreases were weighed and stored in formaldehyde solution for hematoxylin and eosin (HE) staining. This study was carried out in strict accordance with the recommendations in the Guide for the Care and Use of Laboratory Animals of the National Institutes of Health. The protocol was approved by the Committee on the Ethics of Laboratory Animals of Xuzhou Medical College (Xuzhou, China). All surgeries were performed under sodium pentobarbital anesthesia, and all efforts were made to minimize the suffering of the mice.

### Blood glucose and pancreas weight index

Blood glucose was measured using the glucose oxidase method with kits purchased from Rongsheng-Biotech Co. Ltd. (Shanghai, China) in accordance with the manufacturer’s instructions. The assay was based on the reaction of 4-aminoantipyrine and phenol with glucose to yield a red complex. The absorbance was measured at 505 nm. The pancreas weight index (mg/g) was the ratio of the weight of the pancreas to the total body weight.

### Enzyme-linked immunosorbent assay (ELISA)

The levels of insulin in the serum were determined by ELISA. Insulin in the serum first combined with mouse insulin monoclonal antibody, prior to combination with streptavidin-horseradish peroxidase; the optical density of the colored immune complex was then measured at 450 nm. The levels of insulin were determined using a mouse insulin kit according to the manufacturer’s instructions (R&D Systems).

### GSH assay

The level of GSH was measured following the method of Beutler with certain modification (18). The determination of GSH was based on the ability of the -SH group to reduce 5,5′-dithiobis(2-nitrobenzoic acid) and form a yellow anionic product whose optical density was measured at 412 nm. The levels of GSH were determined using the GSH kit from Nanjing Jiancheng Bioengineering Institute.

### Determination of T-AOC

The plasma T-AOC was determined using a modification of the ferric reducing ability of plasma (FRAP) assay reported by Benzie and Strain (19). FRAP reagent was prepared using acetate buffer, ferric chloride and 2,4,6-tripyridyl-S-triazine. The plasma was mixed with FRAP reagent thoroughly prior to determination of the absorbance at 593 nm according to the manufacturer’s instructions (Nanjing Jiancheng Bioengineering Institute).

### HE staining

Pancreatic tissues were harvested from the sacrificed mice and fixed in 10% neutral buffered formalin solution, prior to dehydration in ethanol and paraffin-embedding. Sections measuring 5-μm thickness were prepared using a rotary microtome and stained with HE dyes for microscopic observation.

### Statistical analysis

Data are expressed as the mean ± standard deviation. Statistical analysis was performed using the paired t-test and one-way analysis of variance with Dunnett’s test. P<0.05 was considered to indicate a statistically significant difference.

## Results

### Effects of PPE and BBPE on body weight

**Figure 1:**
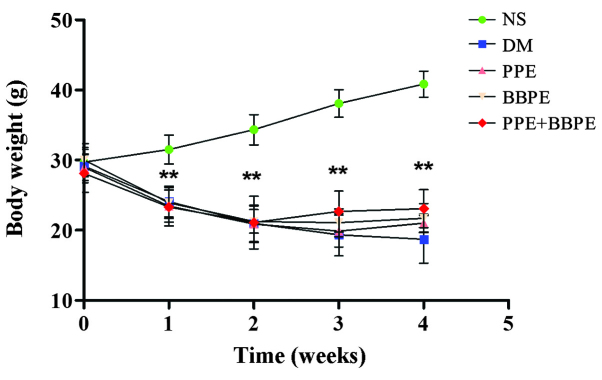
Changes in the body weight of mice. The mice were grouped as follows: NS, normal mice treated with 1% CMC solution; DM, DM mice treated with 1% CMC solution; PPE, DM mice treated with 400 mg/kg PPE; BBPE, DM mice treated with 400 mg/kg BBPE; and PPE + BBPE, DM mice treated with 200 mg/kg PPE plus 200 mg/kg BBPE. Data are presented as the mean ± standard deviation; n=6–9. ^**^P<0.01 vs. the NS group. CMC, carboxymethyl cellulose; DM, diabetes mellitus; PPE, pomegranate peel extract; BBPE, black bean peel extract.

The experimental mice were weighed between weeks 0 and 4 following treatment with PPE, BBPE or PPE + BBPE. The time-point when the development of DM was noted in the mice was recognized as week 0. The body weights of all diabetic mice (the DM, PPE, BBPE and PPE + BBPE groups) were decreased following STZ induction compared with those in the NS group (P<0.01). The body weights of the diabetic mice started to increase following three weeks of treatment with BBPE or PPE + BBPE, but no statistical significance was found (Fig. 1).

### Effects of PPE and BBPE on pancreas weight index and fasting blood glucose and insulin levels

In this study, a mouse model of DM was established by a single intraperitoneal injection of STZ. The pancreas weight index and fasting blood glucose and insulin levels were determined once the mice had been sacrificed (Table I). The levels of fasting blood glucose of all diabetic mice (the DM, PPE, BBPE and PPE + BBPE groups) were significantly increased when compared with those in the NS group. After four weeks of treatment with PPE, BBPE or PPE + BBPE, the levels of fasting blood glucose in the DM mice were reduced. The level of blood glucose in the PPE + BBPE group was lower than that in the PPE group. However, no significant differences were found in the levels of blood glucose, insulin and pancreas weight index between the PPE+BBPE group and the BBPE group (P>0.05). These results demonstrated that PPE and BBPE, respectively, caused significantly inhibitory effects on fasting blood glucose, while the effects subsequent to treatment with PPE + BBPE were stronger than those following PPE treatment alone.

The results also indicated that the insulin levels of all diabetic mice were notably decreased when compared with those in the NS group. Following treatment with PPE, BBPE or PPE + BBPE, the quantities of insulin were increased; however, significant changes relative to the DM group were only found in the PPE + BBPE group (P<0.01). In addition, the level of insulin was significantly increased in the PPE + BBPE group compared with that in the PPE group. These results demonstrated that the combination of PPE and BBPE was more potent in stimulating the secretion of insulin than treatment with PPE alone.

The pancreas weight index in the DM group was decreased (P<0.01) in comparison with that in the NS group. Following treatment with PPE, BBPE or PPE + BBPE, the pancreas weight index was increased compared with that in the DM group. However, statistical significance was only found in the PPE + BBPE group (P<0.05). The combination of PPE and BBPE therefore demonstrated the stronger capacity in increasing the weight of the pancreas in DM mice.

### Antioxidant properties of PPE and BBPE

**Figure 2:**
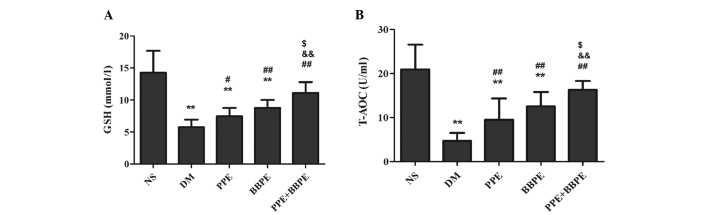
Changes in the (A) GSH levels and (B) T-AOC in mice. The mice were grouped as follows: NS, normal mice treated with 1% CMC solution; DM, DM mice treated with 1% CMC solution; PPE, DM mice treated with 400 mg/kg PPE; BBPE, DM mice treated with 400 mg/kg BBPE; and PPE + BBPE, DM mice treated with 200 mg/kg PPE plus 200 mg/kg BBPE. Data are presented as the mean ± standard deviation; n=6–9. ^**^P<0.01 vs. the NS group; ^##^P<0.01 and ^#^P<0.05 vs. the DM group; ^&&^P<0.01 vs. the PPE group; ^$^P<0.05 vs. the BBPE group. CMC, carboxymethyl cellulose; DM, diabetes mellitus; PPE, pomegranate peel extract; BBPE, black bean peel extract; GSH, glutathione; T-AOC, total antioxidative capability.

The levels of GSH and the T-AOC can reflex antioxidant activity. The levels of GSH and the T-AOC in the DM group were significantly reduced compared with those in the NS group, indicating the lowered antioxidant activity of DM mice. After the four-week treatment period, the values of the two indices were increased in the three treatment groups compared with those in the DM group. Furthermore, the two antioxidant indices in the PPE + BBPE group were significantly higher than those in the PPE or BBPE groups (Fig. 2). The results indicated that PPE and BBPE significantly enhanced the antioxidant capacity in DM mice, while the effect of PPE + BBPE was superior.

### Changes in the pancreatic islets

**Figure 3:**
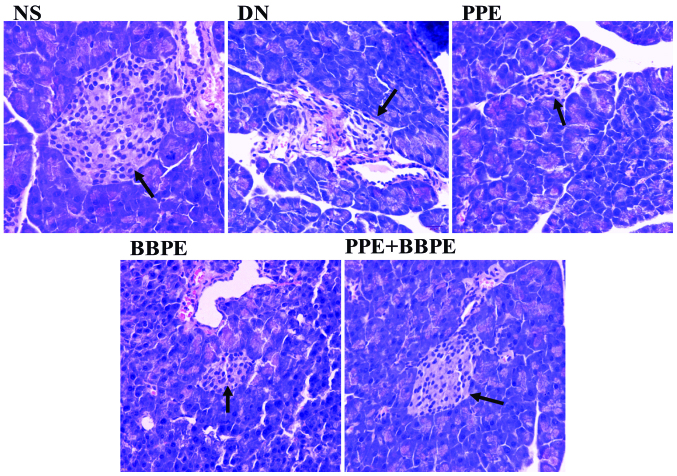
Morphological changes of the pancreatic islets by hematoxylin and eosin staining (original magnification, ×200). The arrows indicate islets. The mice were grouped as follows: NS, normal mice treated with 1% CMC solution; DM, DM mice treated with 1% CMC solution; PPE, DM mice treated with 400 mg/kg PPE; BBPE, DM mice treated with 400 mg/kg BBPE; and PPE + BBPE, DM mice treated with 200 mg/kg PPE plus 200 mg/kg BBPE. CMC, carboxymethyl cellulose; DM, diabetes mellitus; PPE, pomegranate peel extract; BBPE, black bean peel extract.

According to the HE staining images, the islets in the NS group were regularly shaped. The cells in the islets were well distributed and cell sizes were uniform. By contrast, the islets in the DM group were small, with irregular outlines and continuity. Vacuolar denaturation, nuclear concentration and lymphocyte infiltration were observed. In addition, the cells in the islets were irregularly shaped and exhibited a messy arrangement, while the structures of the cells were indistinct. Following treatment with PPE, BBPE or PPE + BBPE, the abnormalities were partially alleviated (Fig. 3).

## Discussion

Diabetes is a chronic disease characterized by disordered metabolism and abnormally high blood glucose levels. The increase in ROS from the mitochondria is deleterious to cellular functioning, and molecules such as hydrogen peroxide and peroxynitrite may cross the mitochondrial membranes and damage macromolecules in other cellular regions (3,20). It has been found that ROS overproduction can directly lead to pancreatic β-cell dysfunction and consequently result in the impairment of insulin secretion in Type 1 diabetes (1). Mammalian cells have a complex network of antioxidant enzymes, including glutathione peroxidases, superoxide dismutase and catalase, and non-enzymatic antioxidants, such as GSH, vitamin C, vitamin E and β-carotene, to scavenge ROS (21). Disturbances in the balance between ROS production and antioxidant defense mechanisms, termed as oxidative stress, result in pancreatic damage in DM (1). GSH acts to protect normal cell structure and function by maintaining the redox homeostasis, quenching free radicals and participating in detoxification reactions. As well as functioning as a direct scavenger of free radicals, GSH is a co-substrate for peroxide detoxification by glutathione peroxidases (16,21). The T-AOC reflects the total antioxidant capacity in the body by its effect of transforming Fe^3+^ into Fe^2+^ (21). In the present study, STZ, an antibiotic produced by *Streptomyces achromogenes*, was used to produce a mouse model of DM through the induction of ROS overproduction and damage to pancreatic β cells (15–17). The entry of STZ into β cells is facilitated via the glucose transporter, type 2 due to the structural resemblance of STZ to glucose (22). Once inside the β cell, oxidative reactions occur with thiol-containing enzymes, such as glucokinase and aconitase, leading to glucose sensing impairments, mitochondrial dysfunction and necrotic cell death (22). In addition, α cells and δ cells in the pancreatic islets undergo necrosis through the influence of STZ (15). The present results showed that there was a fall in the levels of GSH and the T-AOC accompanied by an increase in blood glucose in the DM mice, which indicated the occurrence of oxidative stress and the success of DM model establishment by STZ induction. Histological findings showed that the islets were irregularly shaped and small and that the structures of the cells were indistinct, which was most likely due to oxidative stress induced-protein modification (15).

The black bean contains phytochemicals, including phenolic compounds, which can provide health benefits to the consumers. It has been found that anthocyanins, which constitute a major flavonoid group, can promote endothelial repair and prevent atherogenesis in diabetic apolipoprotein E-deficient mice (23). Furthermore, a higher consumption of anthocyanins and anthocyanin-rich fruit was found to be associated with a lower risk of type 2 diabetes (24). Studies have shown that black seed peel exhibits considerably higher total phenolic indices (including anthocyanins) and antioxidant activities than whole or dehulled black beans, despite the different cultivars of black bean (10,24). The present study therefore used the extracts from black bean peel in DM mice, in addition to a second extract derived from pomegranate peel. Pomegranate peel is rich in antioxidants of the polyphenolic class, which includes flavonoids such as ellagitannins and anthocyanins (11). It has been suggested that ellagitannins could be responsible for the promising antioxidant and antimutagenic activities of PPE (25). Fawole *et al* (26) found that PPE exhibited strong antibacterial and antioxidant activities. Furthermore, epidemiological studies have demonstrated that the consumption of foods rich in flavonoids (the primary polyphenolic compounds) could protect against human diseases associated with oxidative stress, such as coronary heart disease and cancer (11–14). Little information, however, is available regarding the application of PPE and BBPE in DM.

In the present study, the effects of polyphenol-rich PPE and BBPE were observed in DM mice. The results showed that, after four weeks of treatment with PPE, BBPE or PPE + BBPE, the DM mice showed, to different degrees, a decrease in blood glucose, increases in insulin secretion and the pancreas weight index, and an increase in antioxidative activity. The results indicated that PPE and BBPE, respectively, protected the pancreatic β cells from STZ-mediated oxidative stress and thereby stimulated the recovery of pancreatic β cells and the increased synthesis and secretion of insulin, consequently leading to adjustments in the level of blood glucose. It could be therefore be concluded that PPE and BBPE possess significant antidiabetic and antioxidant potential in STZ-induced diabetic mice. The histological observation of the pancreatic tissues further proved the potential protective effects of PPE and BBPE on pancreatic tissue and showed that the protective effects were stronger in the DM mice that were subjected to the joint intervention of PPE + BBPE. This may have been due to the fact that the biological activity of flavonoids depends on the types of phytochemical constituents, the complexity of their structures and the composition of the flavonoid mixtures (27,28), which produce an additive or synergistic effect (29,30). There was, however, no significant change in the body weight following the treatment with the two extracts and their mixture, although an increasing trend was noted; this requires further investigation.

In conclusion, the present study demonstrates that PPE and BBPE, particularly the combination of the two, have the ability to ameliorate hyperglycemia by inhibiting oxidative stress-induced damage to the pancreas; as such, these extracts may be useful in the prevention and treatment of DM. Further studies are required to identify the molecular mechanism involved in the protection of pancreatic tissue by PPE and BBPE in oxidative stress-induced DM mice.

## Figures and Tables

**Table I:** Effects of PPE and BBPE on the blood glucose, insulin levels and pancreas weight index of mice.

Group	n	Blood glucose (mmol/l)	Insulin (mIU/l)	Pancreas weight index (mg/g)
NS	9	6.69±1.06	6.87±0.69	6.03±0.49
DM	8	25.76±4.60^a^	3.73±1.21^a^	5.37±0.40^a^
PPE	6	20.90±3.61^a^,^b^	4.67±1.91^a^	5.74±0.44
BBPE	6	19.55±3.10^a^,^b^	4.96±1.27^a^	5.48±1.30
PPE+BBPE	6	16.72±2.57^a^,^c^,^d^	5.48±1.69^c^,^e^,^f^	5.86±0.57^b^

The mice were grouped as follows: NS, normal mice treated with 1% CMC solution; DM, DM mice treated with 1% CMC solution; PPE, DM mice treated with 400 mg/kg PPE; BBPE, DM mice treated with 400 mg/kg BBPE; and PPE + BBPE, DM mice treated with 200 mg/kg PPE plus 200 mg/kg BBPE. Data are presented as the mean ± standard deviation.

a P<0.01 vs. the NS group;

b P<0.05 and

c P<0.01 vs. the DM group;

d P<0.05 vs. the PPE group; and

e P<0.05 vs. the NS group;

f P<0.01 vs. PPE; The levels in all the groups were compared to those in the BBPE group and no significant differences were found.

CMC, carboxymethyl cellulose; DM, diabetes mellitus; PPE, pomegranate peel extract; BBPE, black bean peel extract.
